# Buffering the impacts of extreme climate variability in the highly engineered Tigris Euphrates river system

**DOI:** 10.1038/s41598-022-07891-0

**Published:** 2022-03-09

**Authors:** Karem Abdelmohsen, Mohamed Sultan, Himanshu Save, Abotalib Z. Abotalib, Eugene Yan, Khaled H. Zahran

**Affiliations:** 1 Geological and Environmental Sciences, Western Michigan University, Kalamazoo, MI USA; 2 Geodynamics Department, National Research Institute of Astronomy and Geophysics, Cairo, Egypt; 3 Center for Space Research, The University of Texas at Austin, Austin, TX USA; 4 Division of Geological Applications and Mineral Resources, National Authority for Remote Sensing and Space Sciences, Cairo, Egypt; 5 Environmental Science Division, Argonne National Laboratory, Lemont, IL USA

**Keywords:** Natural hazards, Hydrology

## Abstract

More extreme and prolonged floods and droughts, commonly attributed to global warming, are affecting the livelihood of major sectors of the world’s population in many basins worldwide. While these events could introduce devastating socioeconomic impacts, highly engineered systems are better prepared for modulating these extreme climatic variabilities. Herein, we provide methodologies to assess the effectiveness of reservoirs in managing extreme floods and droughts and modulating their impacts in data-scarce river basins. Our analysis of multiple satellite missions and global land surface models over the Tigris-Euphrates Watershed (TEW; 30 dams; storage capacity: 250 km^3^), showed a prolonged (2007–2018) and intense drought (Average Annual Precipitation [AAP]: < 400 km^3^) with no parallels in the past 100 years (AAP during 1920–2020: 538 km^3^) followed by 1-in-100-year extensive precipitation event (726 km^3^) and an impressive recovery (113 ± 11 km^3^) in 2019 amounting to 50% of losses endured during drought years. Dam reservoirs captured water equivalent to 40% of those losses in that year. Additional studies are required to investigate whether similar highly engineered watersheds with multi-year, high storage capacity can potentially modulate the impact of projected global warming-related increases in the frequency and intensity of extreme rainfall and drought events in the twenty-first century.

## Introduction

The response of hydrologic systems to global warming has been a major topic of research and debate by the scientific community over the past two decades^1–4^. Under global warming conditions, the global long-term trends in average precipitation show an increase of 7%/°C in atmospheric capacity to hold water associated with 2%/°C increase in global mean precipitation^5^.

Analysis of multiple global and regional climate models^2,6^ shows a projected decrease in light to moderate events (0.1 mm/h < PPT ≤ 2.0 mm/h) and an increase in intensity and frequency of heavy precipitation (PPT) (2 mm/h < PPT ≤ 10 mm/h) and very heavy PPT (> 10 mm/h) events. The projected increase in heavy and very heavy precipitation events will undoubtedly increase flood risks on a global scale^7^. Flooding events between 1980 and 2016 left behind more than 225,000 fatalities and economic losses exceeding $1.6 trillion^8^, and those losses are expected to increase by up to a factor of 20 by the end of the twenty-first century if no action is taken to reverse the course^9^. Global flood risk projections from climate models reveal significant increases in flood frequency by the end of the twenty-first century in Southeast Asia, the Indian Subcontinent, eastern Africa, and the northern half of the Andes, as well as the highlands in Iran and Turkey, the source areas of the Tigris and Euphrates rivers^10^.

In contrast, precipitation over other regions will remain unchanged or decline significantly^1,11,12^. Climatic projections show a decrease, or no significant change, in drought frequency over the northern high latitudes, eastern Australia, and eastern Eurasia^12,13^, whereas the southwestern US and the Rocky Mountain^14^, north and central Africa^15^, the Sahel zone^16^, Amazonia, and Northeast Brazil will witness a reduction in precipitation by up to 40%^17^. Projections of river discharge extremes (up to the year 2100) using simulated daily river discharge derived from high-resolution general circulation models (between the years 1901 and 2000) indicated that under global warming conditions, the frequency and intensity of droughts is expected to increase globally^12^.

The projected intensification of flooding and drought events in the twenty-first century could occur within the same region. It was suggested that a + 2 °C global warming will produce extreme floods and severe and extended droughts in western and southern Europe including Spain, France, Italy, Greece, the Balkans, the south of the UK, and Ireland^18^. Similarly, the Niger, Ganges, and Congo River basins could witness increases in both flood and drought frequencies^12^. More than ever, there is a need to regulate and manage the projected extreme flooding and drought events.

Dams mitigate the destructive impacts of floods, store excess water in wet seasons/periods, and regulate its consumption during dry seasons/years^19^. At present, some 58,000 large dams (dam height > 15 m) regulate the flow in more than 50% of the Earth’s river systems^20,21^. Recent flood modeling studies have shown that dams reduce the exposure of the world’s population to floods by as much as 20.6%^22^. The role of dams in drought mitigation and sustenance of water supplies through storage and controlled distribution has been recognized in ancient and historical periods, and is more so in modern times^23^ (see Supplementary Notes). The performance of reservoirs under climate variability has been widely examined using various modeling approaches such as General Circulation Models^24^, Community Earth System Model Large Ensemble^25^, data-driven behavioral modeling^26^, Ensemble Forecast Operations^27^, and sociohydrological models^28^. The application of the majority of these models require intensive data on reservoir characteristics as well as historical flow conditions. Such data are not available for many of the river basins worldwide where water sharing rights among riparian countries are contested and sharing data is not honored. The lack of agreed-upon arrangements between riparian countries for managing water shares and reservoir operation schemes (e.g., Nile River^29^; Yaluzangbu-Brahmaputra River^30^; Tigris-Euphrates basins^31^) calls for unconventional methods to examine the reservoir performance under a changing climate.

Gravity Recovery and Climate Experiment (GRACE) and GRACE Follow-On (GRACE-FO) solutions, together with data from other satellites (e.g., Global Precipitation Measurement (GPM) mission, Tropical Rainfall Measuring Mission (TRMM), Sentinel-1, and Landsat) and in-situ data (GPS, geochemical, and hydraulic head data) have been used widely to monitor spatial and temporal variations in water storage in response to climate change and anthropogenic activities^32^. Many of those applications were devoted to monitoring basin-scale flood potential (e.g., Missouri River basin^33^; Ganges–Brahmaputra Delta^34^; Yangtze River basin^35^; Red River basin^36^; Mackenzie River basin^37^; Liao River basin^38^, and the Nile River basin^39,40^).

With few exceptions^41–43^, investigating the impact of dams and associated artificial reservoirs, hereafter referred to as reservoirs, on water storage received less attention, primarily due to the coarse resolution of GRACE data (spatial resolution of ~ 400 km) and the relatively small size of reservoirs^35^. Even fewer studies targeted multiple small reservoirs (< 40 km^3^) within a basin (Tigris-Euphrates Basin^44^; Jinsha River Basin^45^). Given the projected increase in the frequency of extreme rainfall and drought events under climate change^2,6^, the large cumulative storage capacity of reservoirs worldwide (7000 to 8300 km^3^)^21^, and the widespread occurrence of data-scarce river basins in almost all continents it is now necessary, more than ever, to develop robust procedures to examine the role dams and their reservoirs could play in buffering the impact of climate variability (i.e., extreme flood and droughts) on the basin scale in data-scarce river basins.

Examples of data scarce basins include the Vrbas River basin in Europe^46^, the Blue Nile River and Wami River basins in north and central Africa^47,48^, the Lower Jordan River, the Kharaa River, and Cau River basins in western, central, and southeast Asia, respectively^49–51^, and the Fragata River and the Upper Paraguay River basins in South America^52,53^.

In this study we address this issue using the Tigris-Euphrates watershed (TEW) as our test site and GRACE solutions and radar altimetry as our prime datasets. We selected the TEW as a test site for the following reasons: (1) it is a highly engineered system (30 dams; total storage capacity: 250 km^3^); (2) the watershed witnessed a severe drought (2007 to 2018) and an impressive recovery in 2019 during the GRACE and GRACE-FO operational period.

### Geological, hydrological, and climatological setting of the TEW

**Figure 1:**
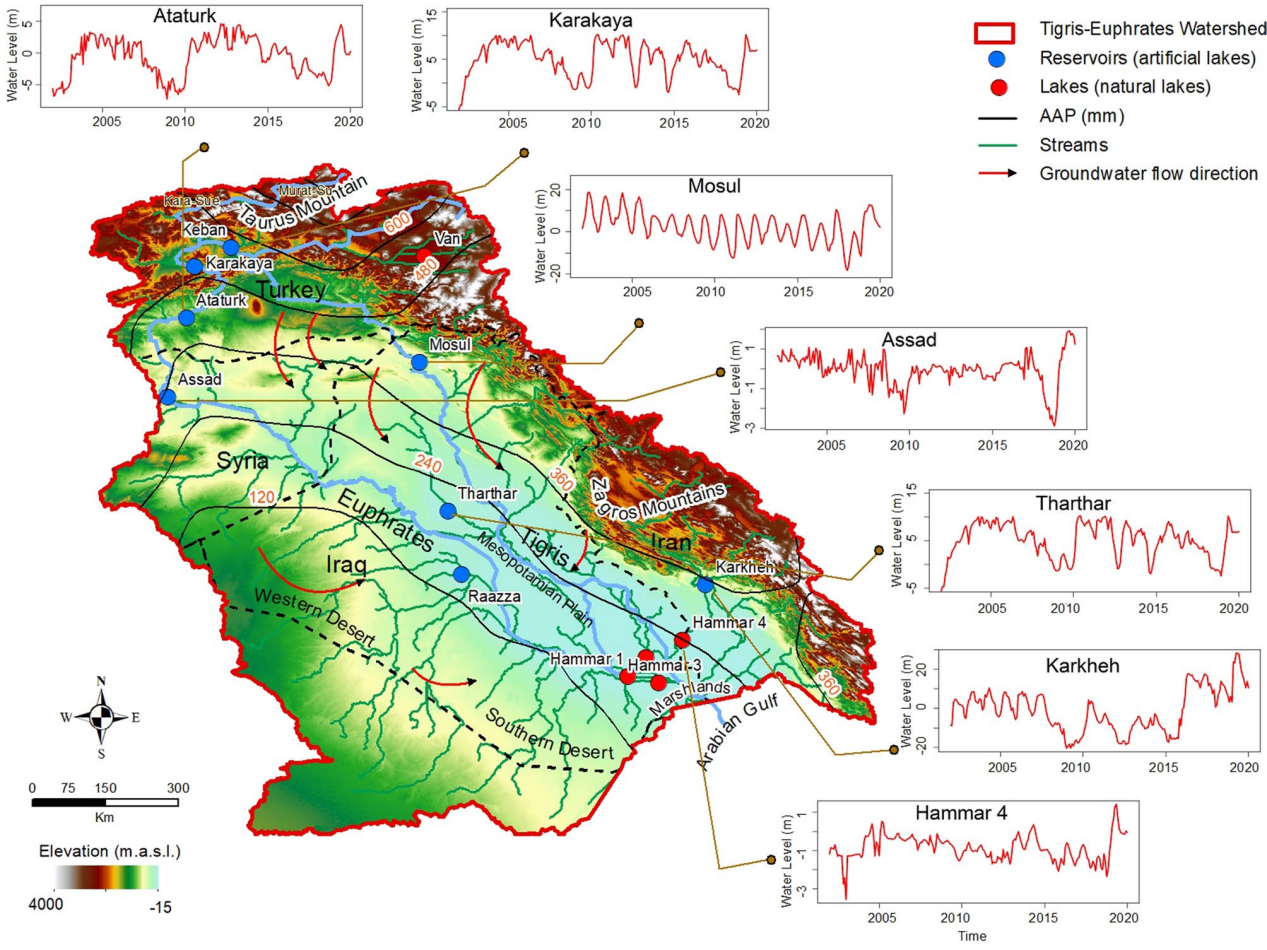
Location map of the TEW. Figure shows the spatial variations in elevation in m.a.m.s.l across the TEW and the distribution of stream networks extracted from Shuttle Radar Topography Mission (SRTM) data using ArcGIS 10.8 hydrological tools (https://www.arcgis.com/). Also shown are the distribution of Tigris and Euphrates rivers and the source areas (Taurus and Zagros Mountains), to the north and east, the deserts to the west (Western Desert) and south (Southern Desert), and the central Mesopotamian Plain. Also shown, the groundwater flow directions^67,93^, the main reservoirs (blue circle), lakes (red circle). Also shown are time series of surface water level variations (2003–2020) from radar altimetry (Global Reservoir and Lake Monitoring (GRLM) database; available at https://www.pecad.fas.usda.gov/cropexplorer/globalreservoir/) over the TEW lakes (e.g., Hammar 4 in Iraq) and reservoirs (Karkheh in Iran, Mosul and Tharthar in Iraq, Karakaya and Ataturk in Turkey, and Assad in Syria) showing a significant rise in water levels following the extreme precipitation event in 2019.

**Figure 2:**
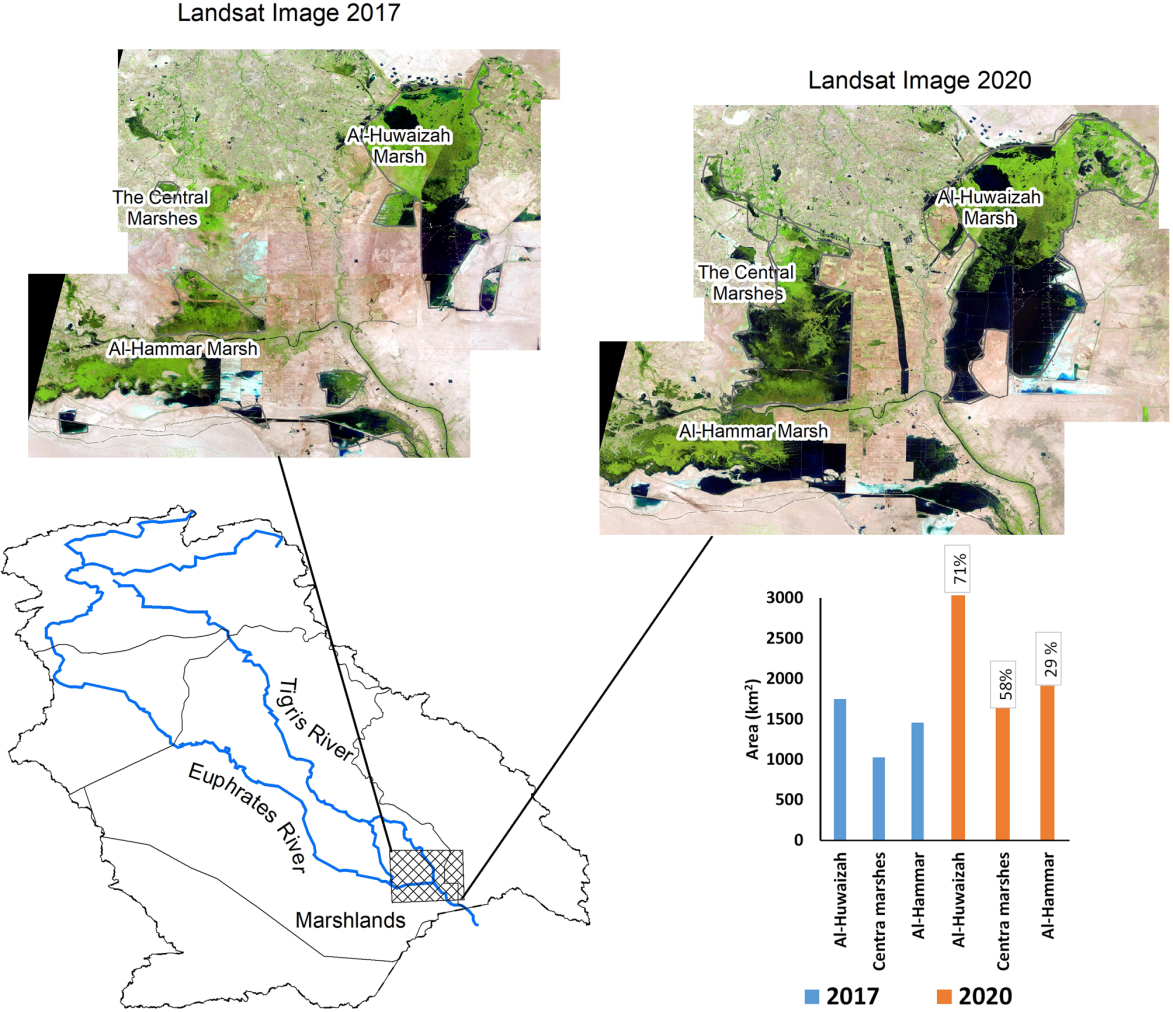
The areal extent of the Mesopotamian marshes before and after the 2019 extreme precipitation event in years 2017 and 2020, respectively. Comparison between the areal extent of the Mesopotamian marshes (Al-Huwaizah, Central, and Al-Hammar) in southern Iraq using false-color composites generated from 30 m multispectral Landsat 8 data (https://www.usgs.gov/) using ArcGIS 10.8 Spatial analyst tools (https://www.arcgis.com/) before and after the extreme 2019 precipitation event.

The TEW covers an area of 1 × 10^6^ km^2^ in Turkey, Syria, Iraq, Iran, and Kuwait^54^. The two main rivers within the watershed—the Euphrates River, the longest in west Asia (length: 2800 km), and the Tigris River (length: 1900 km) originate from the highlands of Turkey, Iran, and Syria^54^. The two rivers (Fig. 1) flow downstream towards the alluvial plain in central Iraq, merge together near Basra, and feed the marshlands in southern Iraq before discharging in the Arabian Gulf (Fig. 2). Approximately, 60% of the river flow within the watershed is carried by the Euphrates and the remaining 40% by the Tigris^55^. The flow originates as snowfall over the highlands during the wet winter season (November–April), snow accumulations melt in the spring, feed the river systems, recharge the aquifers, and sustain the livelihood of large sectors of the population, especially those downstream^54,56^. The average monthly temperature of the TEW ranges from 6 to 16 °C in the wet season and much higher temperatures (up to 43 °C) during the dry summers (May–October), causing evaporation and minimal infiltration of precipitation and recharge to aquifers during these summer periods^57,58^.

The precipitation over the highlands varies considerably from one year to the next, causing large variations in the Tigris and Euphrates river discharge and drought and flooding conditions across the watershed. The annual discharge of the Euphrates was 16.8 km^3^ in 1961, 43.4 km^3^ in 1963, and 53.5 km^3^ in 1969^55^. A similar pattern was reported for the Tigris; the discharge was 7.9 km^3^, 31 km^3^, and 34.3 km^3^ in years 1961, 1963, and 1969, respectively^55,59^. Historical records report extreme drought conditions in years 1929 and 1930, when the Euphrates River flow dropped down to 10.7 km^3^/year. The high floods of the Euphrates destroyed the entire city of Nineveh in 612 B.C.E., and in 1896, the Tigris River rose by up to 3 m in one night, destroyed embankments, and flooded large sectors of Baghdad^60^. This large variability in precipitation and river discharge^42–44^ was mitigated to a large extent by aggressive engineering programs involving construction of many major dams over the Tigris and Euphrates rivers starting in mid-1970s and extending into the twenty-first century.

The largest of the constructed dams along the Euphrates are the Ataturk in Turkey (area: 817 km^2^; storage capacity 48.7 km^3^), the Keban in Turkey (area: 675 km^2^; storage capacity: 31 km^3^), and the Raazza in Iraq (area: 1810 km^2^; storage capacity: 26 km^3^). The largest reservoirs on the Tigris are Karakaya (area: 268 km^2^; storage capacity: 9.5 km^3^) in Turkey and Tharthar (area: 2170 km^2^; storage capacity: 72.8 km^3^) and Mosul (area: 380 km^2^; storage capacity: 11.1 km^3^) in Iraq^58^ (Fig. 1).

The TEW climate is continental subtropical in the northern upstream regions and highlands and arid to semiarid in the southern downstream regions^61^. The climatic variability described above over the TEW and over large sections of Europe and the Middle East in general is largely related to, or correlated with, the North Atlantic Oscillation (NAO), Mediterranean Oscillation Index (MOI), El Nino Southern Oscillation (ENSO)^61^, or the sea surface temperature (SST) anomalies that represent the intensity of these climatic oscillations^62^ (see Supplementary Notes).

We accomplish the following over the study area: (1) identify the wet and dry periods from the Global Precipitation Climatology Centre (GPCC), a monthly combined satellite-gauge precipitation dataset (1920–2020), and from the non-seasonal terrestrial water storage (TWS) from GRACE_TWS_ and GRACE-FO_TWS_ datasets, hereafter referred to as GRACE_TWS_, over the period 2001–2021; and (2) develop innovative procedures that utilize multi-mission satellite radar altimetry (e.g., TOPEX/Poseidon, Envisat, and Jason-1/2/3) and multi-sensor data (Landsat 5, 7, and 8) to construct extended surface water level data over the TEW, fill data gaps in the radar altimetry time series, and use these methods to measure with accuracy the temporal variations in surface water storage (SWS) over the individual TEW reservoirs and lakes. Satellite-based observations were adopted given the paucity, and in some cases, the absence of direct water level measurements and stage storage curves over the TEW reservoirs and lakes. Finally, we demonstrate an impressive recovery of the system following a prolonged (2007–2018) drought by an extreme precipitation (1 in 100 years) event in 2019 enabled largely by the impoundment of a large portion of the runoff within the reservoirs.

## Results

### Temporal variations in GRACE_TWS_

**Figure 3:**
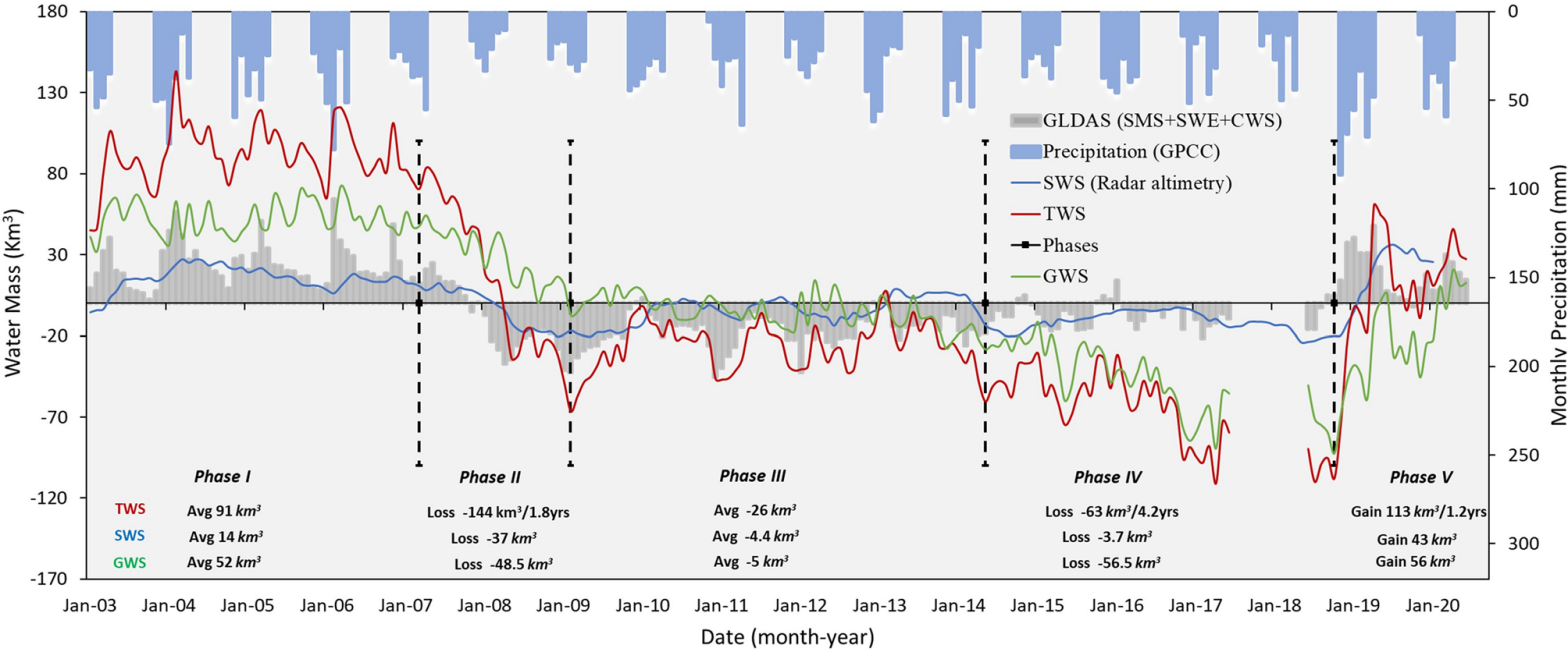
Comparisons between the time series of the TWS_GRACE_, SWS_ALT_, GWS_GRACE_, (SMS + SWE + CWS)_GLDAS_, and seasonal precipitation. Time series were derived over the TEW for each of the investigated time periods (Phases I–V). The comparisons are made in units of monthly variations in water mass averaged over the TEW.

The non-seasonal GRACE terrestrial water storage time series over the TEW (Fig. 3 and Table 1) shows significant variations throughout the investigated period (2003 to 2020). Five phases were identified over the past two decades. The watershed witnessed positive (average GRACE_TWS_: 91 km^3^) and near-steady GRACE_TWS_ values (5.6 ± 4 mm/4.2 year; 6.3 ± 5 km^3^/4.2 year) in years 2003 through 2007 (Phase I) followed by a sharp decline and significant losses in GRACE_TWS_ (− 130 ± 4 mm/1.8 year; − 144 ± 5 km^3^/1.8 year) in years 2007–2009 (Phase II). The period from 2009 to 2014 (Phase III) is characterized by negative (average GRACE_TWs_: − 26 ± 7 km^3^) and near-steady GRACE_TWS_ values (2.8 ± 6 mm/5.1 year; 3.1 ± 7 km^3^/5.1 year), followed (2014–2018; Phase IV) by a second decline in GRACE_TWS_ and additional losses (− 57 ± 7 mm/4.2 year; − 63 ± 8 km^3^/4.2 year) to the system. This continuous and long-term depletion of the system throughout periods II, III, and IV was reversed in 2019 by an impressive recovery of the Tigris-Euphrates hydrologic system, as evidenced by the increase in GRACE_TWS_ by as much as 101 mm or 113 km^3^ in a single year (2019). Our analysis has shown that by 2018 (the end of phase IV), the system had lost a total of 204 km^3^ largely during phases II and IV, but recovered 50% of those losses in 2019 and retained these gains in 2020, an observation that could signal the beginning of a positive and near-steady Phase V.

**Table 1:** Partitioning of TWS_GRACE_ over TEW. TWS_GRACE_, SWS_ALT_, (SMS + SWE + CWS)_GLDAS_, and GWS_GRACE_ trends over the TEW for each of the investigated time periods (Phases I–V).

Phase	Years	ΔTWS^a^		ΔSWS^b^		Δ(SMS + SWE + CW)^c^		ΔGWS^d^	
		(mm/year)	(km^3^)	(mm/year)	(km^3^)	(mm/year)	(km^3^)	(mm/year)	(km^3^)
Phase I	4.2	5.7 ± 4	6.3 ± 5	1.7 ± 0.4	1.9 ± 0.5	1.1 ± 6	1.17 ± 7	2.9 ± 7	3.2 ± 9
Phase II	1.8	− 130 ± 4	− 144 ± 5	− 33.5 ± 3	− 37 ± 3.4	− 52.6 ± 4	− 58.5 ± 5	− 43.8 ± 6	− 48.5 ± 8
Phase III	5.1	2.8 ± 6	3.1 ± 7	14.5 ± 0.4	16 ± 0.5	5.6 ± 4	6.2 ± 5	− 17.2 ± 7	− 19.1 ± 9
Phase IV	4.2	− 57 ± 7	− 63 ± 8	− 3.3 ± 0.7	− 3.7 ± 0.8	− 2.53 ± 4	− 2.8 ± 4	− 51.1 ± 8	− 56.5 ± 9
Phase V	1.2	101 ± 9	113 ± 11	39 ± 3.7	43 ± 4	12.7 ± 8	14 ± 9	49.3 ± 13	56 ± 15

GRACE observations, GLDAS outputs, and radar altimetry measurements were used to estimate the partitioning of TWS in GWS.

^a^ΔTWS: Change in terrestrial water storage.

^b^ΔSWS: Change in surface water storage over the 13 main reservoirs and lakes.

^c^Δ(SMS + SWE + CW): Change in soil moisture storage + snow water equivalent + canopy water.

^d^ΔGWS: Change in groundwater storage.

### Temporal variations in Average Annual Precipitation (AAP)

We examined the AAP and monthly precipitation from GPCC data over the TEW to investigate whether the observed interannual variations in GRACE_TWS_ during phases I through V could be related to temporal variations in precipitation throughout the GRACE and GRACE-FO period (2003–2020), and to examine whether the patterns of precipitation during this period deviated from those in the previous years (1920–2000).

**Figure 4:**
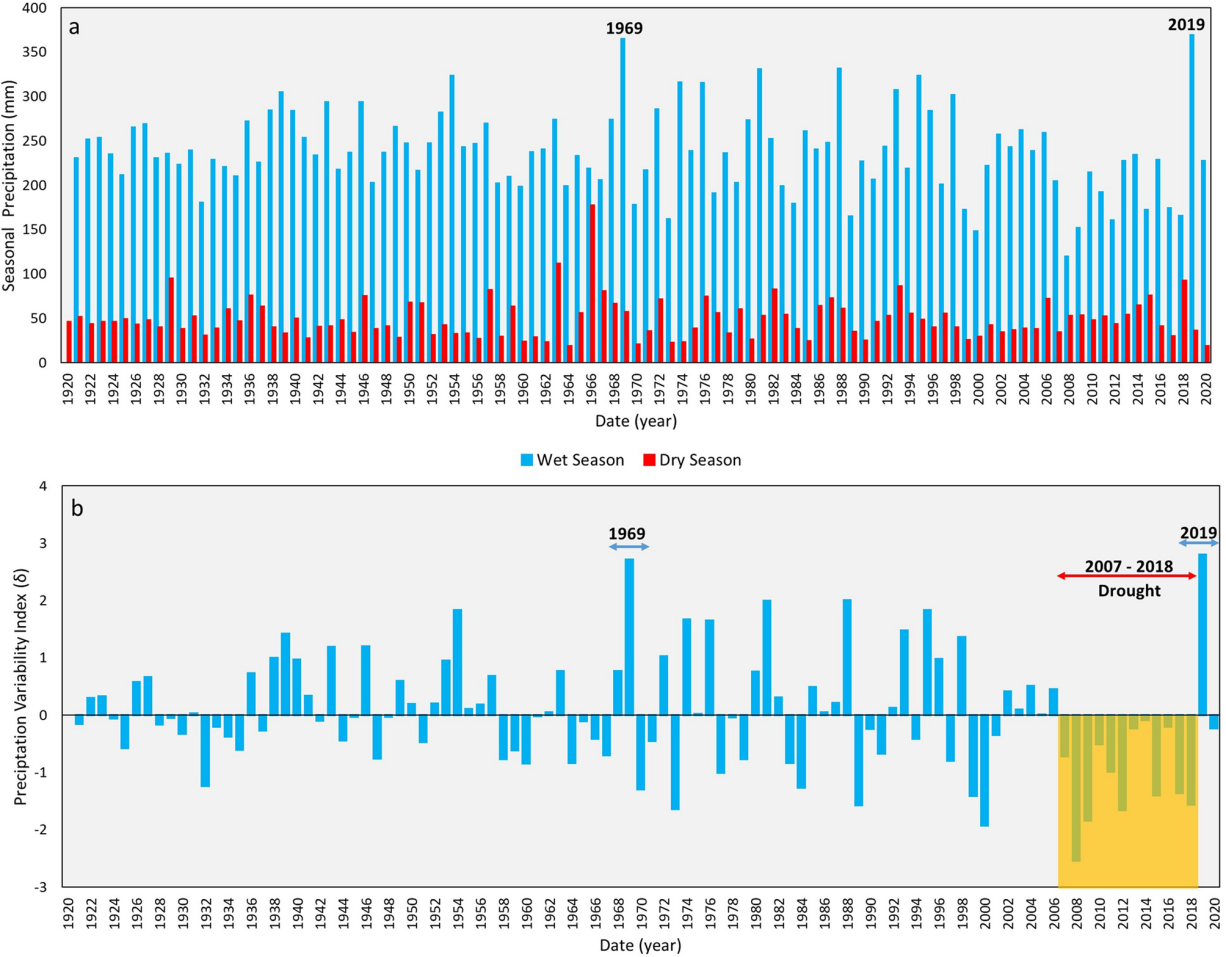
Precipitation and variability index (δ) time series derived from GPCC data (1920–2020). (**a**) Comparison between precipitation during wet seasons (winter and spring: November–April; blue columns) and dry season (summer: May–October; red columns) showing much higher precipitation rates during the wet seasons. (**b**) Use of variability index (δ) time series to identify the drought (− δ) and wet (+ δ) periods; the figure shows a severe and prolonged drought (2007–2018; highlighted in yellow) and the wettest years (highest index values) in 1969 and 2019.

Examination of the precipitation during the wet and dry seasons reveals that the former (avg: 237 mm/year) far exceeds the latter (avg: 48 mm/year) (Fig. 4a). Most of the precipitation in the dry summer season ends up as losses to evaporation given the high summer temperatures and the limited precipitation during the summers. In contrast, minimal losses to evaporation occur during the wet winter and spring seasons during which accumulation and melting of snow occurs^54,57,58^. Thus, it is the wet winter and snow melting seasons that drive the TEW hydrologic system^54,56^.

A comparison between the GRACE_TWS_ and the seasonal precipitation (wet season) represented by AAP datasets throughout the periods covered by phases I through V reveals high correspondence (Fig. 3). The figure shows a severe decline in GRACE_TWS_ values in phase II (− 144 ± 5 km^3^/1.8 year; − 80 km^3^/year) and a moderate decline in Phase IV (− 63 ± 8 km^3^/4.2 year; − 15 km^3^/year). Similar patterns were observed for precipitation, where the AAP of 534 km^3^ in Phase I was reduced to 330 km^3^ in Phase II, a 38% reduction, and the AAP of Phase III (451 km^3^) was reduced to 411 km^3^, a 12% reduction in Phase IV. A significant increase in GRACE_TWS_ (113 ± 11 km^3^/2.2 year; 51 km^3^/year) in Phase V correlated with a dramatic increase (70%) in AAP (Phase IV: 411 km^3^; Phase V: 697 km^3^) during the same period. This increase in precipitation in Phase V is largely due to extreme precipitation in the spring of year 2019.

The frequency of recurrence of the 2019 extreme precipitation event and droughts over the study area was investigated by examining the long-term (1920–2020) GPCC precipitation record (Fig. 4a). Inspection of Fig. 4a reveals that the 2019 AAP (653 mm; 726 km^3^) is the highest during the GRACE and the GRACE-FO periods; it exceeded the AAP (388 mm) in the remaining years (2003 to 2018) by over 68%. Not only was 2019 an anomalous year throughout the GRACE and GRACE-FO mission years, but it was the highest in a century. A similar yet slightly smaller AAP (628 mm; 699 km^3^) was reported in 1986, making the precipitation in 2019 a 1 in 100-year event. Examination of the precipitation variability index (Fig. 4b) reveals a prolonged 12-year drought that extended from 2007 to 2018, the longest throughout the past 100 years. The extreme precipitation events in 1969 and 2019 display the highest precipitation variability index (δ: + 2.7 and + 2.8, respectively).

### Temporal variations in lakes and reservoirs area and surface water levels

The anomalous and extreme precipitation event in 2019 must have had an impact on the watershed’s surface water systems, namely its natural lakes, reservoirs behind dams, and marshes. Figure 2 shows the variations in the area covered by the Al-Huwaizah, Central, and Al-Hammar marshes in Iraq, where the surface area increased from 4238 km^2^ in 2017 to 6530 km^2^ in 2020, an increase of 54%. Not only did the area of the marshes increase, but the area covered by surface water within the marshes, hereafter referred to as lakes, and the surface water levels of these water bodies, increased as well.

Figure 1 shows the variations in surface water level from radar altimetry over several lakes and reservoirs within the watershed. The Hammar 4 Lake (Iraq) rose by 2 m, the Karkheh reservoir (Iran) by 16 m, the Mosul and Tharthar reservoirs (Iraq) by 12 and 15 m, respectively, the Karakaya and Ataturk reservoirs (Turkey) by 11 and 8 m, respectively, and the Assad reservoir (Syria) by 4 m.

The temporal and spatial variations in SWS from radar altimetry data (SWS_ALT_) were estimated over the TEW; these variations are largely controlled by storage in reservoirs compared to lakes; the contributions to these variations are 93 to 7% in favor of the former (reservoirs). The total contribution from both rivers to the SWS in the TEW was found to be small compared to that from the reservoirs (< 6% SWS_ALT_). As such, and given the discontinuous and limited radar data over the Tigris and Euphrates, their contributions to the SWS were ignored. The estimated SWS_ALT_ was compared to the variations in GRACE_TWS_ and in groundwater storage (GRACE_GWS_) throughout the five phases (Fig. 3). Figure 3 shows two significant features. First, the variations in SWS_ALT_ within the individual phases and between the phases are modest compared to those observed in the GRACE_TWS_ and GRACE_GWS_ time series. This is to be expected given that the construction of dams and their reservoirs is intended in the first place to modulate the interannual variations in precipitation and runoff, making the SWS_ALT_ less sensitive to interannual climatic variabilities compared to GRACE_TWS_ and GRACE_GWS_. Visual inspection of Fig. 3 shows small interannual variations within the SWS_ALT_ time series compared to those of the GRACE_TWS_ and GRACE_GWS_ series, as evidenced by the smaller standard deviation of in SWS_ALT_ across the entire period covered by Phases I through V compared to GRACE_TWS_ and GRACE_GWS_ (standard deviation: SWS_ALT_ 14 km^3^; GRACE_TWS_ 61 km^3^; GRACE_GWS_ 39 km^3^) for the same period.

In watersheds lacking reservoirs, most, but not all, of the runoff ends up as losses from the watershed water budget that are carried out of the system by river networks. In our case that would have been the discharge of the TEW into the Gulf prior to 1950, when the system had virtually no storage capacity to capture any of the runoff, which amounted to 80 km^3^ on the average^54,58^. With the progressive construction of major dams that started in the 1970s and extended into the twenty-first century, large portions of the runoff carried by the TEW river network were being intercepted by, and impounded behind, the dams in reservoirs. In 2019, the TEW received a 1 in 100-year extreme precipitation event, and by that time the total storage capacity of the system had increased to 250 km^3^ and was capable of capturing large proportions of precipitation and runoff. The precipitation in 2019 resulted in a dramatic increase in storage across the watershed, a gain of 113 ± 11 km^3^ in GRACE_TWS_, about 38% of which (43 km^3^) was captured in the reservoirs.

### Temporal variation in groundwater storage and recharge from lakes

The time series of groundwater storage (GWS) over the TEW (Fig. 3) was calculated using GRACE_TWS_ and Global Land Data Assimilation System (GLDAS)-derived soil moisture storage (SMS), snow water equivalent (SWE), and canopy water storage (CWS) and radar altimetry-derived SWS. Inspection of Fig. 3 and Table 1 reveal positive (average GRACE_GWS_: 52 ± 9 km^3^) and near-steady GRACE_GWS_ values (trend: 3 ± 7 mm/4.2 year, 3 ± 9 km^3^/4.2 year) during Phase I, followed by a decline (trend: − 43.8 ± 6 mm/1.8 year) and significant losses in GRACE_GWS_ (− 48 ± 8 km^3^/1.8 year). The period from 2009 to 2014 (Phase III) is characterized by negative (average GRACE_GWS_: − 5 km^3^) and near-steady GRACE_GWS_ values (− 17 ± 7 mm/5.1 years; − 19 ± 9 km^3^/5.1 years), and the following 4 years (2014–2018; Phase IV) by a second decline in GRACE_GWS_ and additional losses (− 51 ± 8 mm/4.2 year; − 56 ± 9 km^3^/4.2 year) to the system (Table 1). Phase V (2018–2020) is characterized by marked recovery in GWS with a total increase of 56 ± 15 km^3^/year that compensated for 45% of the total GWS losses during the dry period (2007–2018).

### Potential climatic drivers

Previous work demonstrated that the climatic variability over the TEW and over large sections of Europe and the Middle East is largely related to, or correlated with, the NAO, MOI, (ENSO^61,63^, or the SST anomalies that represent the intensity of these climatic oscillations. We correlated the temporal variations of these indices with the AAP to examine which of these indices and parameters correlated best with the identified extreme precipitation and drought events (see Supplementary Fig. S1). During the extreme precipitation event of 2019 (AAP: 726 km^3^), the NAO oscillation index (− 1.7) and its SST-based index (− 0.3) were low, whereas during the drought years (2007 to 2018), the SST-based indices of both the SAO and NAO were high (average: SAO: 0.4; NAO: 0.4) (see Supplementary Fig. S1). Additional rigorous statistical analyses should be conducted to unravel the complexity of the interactions between these oscillations and parameters and their impacts on precipitation over the TEW.

## Summary and discussion

The projected increase in the frequency and intensity of extreme rainfall and drought events in the twenty-first century due to climate variability associated with global warming will impact many of the major world’s watersheds. We may already be observing these effects over the TEW; the watershed witnessed a prolonged (2007 to 2018) and intense drought (AAP < 400 km^3^) that had no parallels over the past 100 years and a 1 in a 100-year extreme precipitation event (AAP: 653 mm; 726 km^3^) in 2019 that ended the drought.

While these climate change-related events will introduce devastating socioeconomic impacts on many of the world’s watersheds and their populations, many of the highly engineered watersheds (e.g. Mississippi in North America, storage capacity: 250 km^3^; Paraná in central South America, storage capacity: 65.8 km^3^)^22^, one of which is the TEW, will be spared. The historical records of the TEW reveal high variability of flow in both the Tigris and Euphrates that caused flooding events across the watershed and disrupted irrigation practices^55,58^. The threat of floods has been minimized with the control of river flow by regulating the discharge from dams in Turkey (average annual discharge (AAD): 48 km^3^/year) and Iraq (AAD: 26.5 km^3^/year), and to a lesser extent by the Syrian (AAD: 3.2 km^3^/year) and Iranian (AAD: 4.7 km^3^/year) dams^58,64^.

In 2019, the TEW received a 1 in 100-year precipitation event that would have caused extreme flooding events if the TEW dams were not in place, and much of the runoff from this wet year would have been lost as river discharge in the Gulf. Instead, some 43 km^3^ of the runoff were apparently captured in the lakes and reservoirs (Fig. 3). This added reservoir storage can be used to maintain adequate stream flow in the TEW river network for years to come, especially the years of low precipitation. The relatively high AAP in Phase I (534 km^3^) compared to phases II (330 m^3^), III (451 km^3^), and IV (411 km^3^) was reflected in the positive and high average annual variations in SWS_ALT_ values (179 km^3^) in Phase I compared to negative variations in phases II (− 53 km^3^), III (− 43 km^3^), and IV (− 139 km^3^). The observed drop in SWS_ALT_ in these three phases (II, III, and IV) is here interpreted to indicate the release of impounded reservoir waters to compensate at least in part for the reduced river flow during these dry years. We suggest that the TEW dams modulated some, but not all, the impacts of the prolonged drought that started in 2007 and ended in 2018; similarly, the impounded SWS_ALT_ in 2019 will be effective in reducing the impacts of dry years in upcoming years.

Not only do the dams impound excess runoff within their reservoirs and add to the watershed’s SWS budget, but they can be significant sources of recharge to the underlying aquifers. The more porous, fractured, or karstic the reservoir bedrock, the larger the discharge from the reservoir and the recharge to the underlying aquifer. One would expect high rates of infiltration and discharge from reservoirs constructed over karstified or fractured bedrocks^32^. Examples include Mosul reservoir (maximum storage capacity: 11.1 km^3^) in northwest Iraq, whose bedrock is formed of karstic gypsum and limestone of the Fatha Formation of Middle Miocene age^65^, and Raazza reservoir (maximum storage capacity: 26 km^3^) in Iraq’s Western Desert, a reservoir floored by highly fractured karstic Miocene carbonates of the Dammam formation with high transmissivity and permeability for groundwater flow^66,67^.

Our findings suggest that the highly engineered TEW watershed is better prepared to deal with the projected increase in the frequency and intensity of extreme rainfall and drought events in the twenty-first century. During the extreme rainfall events, the TEW system captures excess runoff, increase the surface and groundwater storage of the watershed, and minimize flooding events. The system modulates water shortages during prolonged and intense droughts through managed release of the captured excess waters.

While highly engineered watersheds could modulate the projected climate change-related extreme floods and droughts in the twenty-first century, we should not lose sight of the negative impacts associated with the development of such highly engineered systems. Construction of high capacity dams on transboundary rivers can cause disputes over water rights between the river basin riparian countries, especially during the filling periods, which may lead to severe socioeconomic instabilities (e.g., the Tigris-Euphrates river basin^68^ and the Nile River basin^69^). Dams impound river flow, create artificial reservoirs, and increase surface water area and losses to evaporation; the global dam-related evaporative losses were estimated at 350 km^3^ in 2010^70^. Dams alter the natural flow of streams, which in turn modify ecological processes, reduce biodiversity (e.g., nutrient cycling^71^), modify river sediment transport^72^, and cause biotic changes in downstream ecosystems^73,74^. The achievement of water supply and flood control objectives can produce unsteady flow regimes along river stretches proximal to dams, which could interfere with the implementation of other significant objectives (e.g., navigation and recreation^75^). Moreover, the over-reliance on impounded reservoir waters could increase the basin vulnerability to droughts on the long-run^76^.

The paradox of building dams, benefits versus drawbacks, is an old one that has been, and will continue to be debated, by researchers from different disciplines and angles. Here we suggest that additional studies are needed to investigate whether similar highly engineered watersheds with multi-year, high storage capacity can potentially modulate the impact of projected global warming-related increases in the frequency and intensity of extreme rainfall and drought events in the twenty-first century. This could be attained by conducting studies similar to the one adopted in this study, especially in data scarce regions. In data rich watersheds, one or more of the following approaches could be adopted: (1) examining paired river basins that have experienced similar flood and drought events with and without highly engineered systems or reservoirs, (2) modeling a river system with and without reservoirs, and (3) modeling several highly engineered river systems, and testing their performance under multiple streamflow regimes. If findings similar to those reported over the TEW were observed, then an additional factor that should be considered, the added capability of highly engineered river system in buffering the impacts of the projected climate extremes in the twenty-first century.

## Methods

We adopted a four-fold methodology throughout the investigated period. We first extracted the temporal and spatial variation in GRACE_TWS_ monthly solutions over the watershed (Task I) and those for the precipitation data from the Global Precipitation Climatology Centre (GPCC) were used to identify extreme events and to examine the degree to which the TWS signal was impacted by the identified extreme precipitation events (Task II). Then we extracted the temporal variations in surface water level and in the volumes of the main reservoirs and lakes (SWS) to examine the degree to which they modulate the impacts of climate variabilities (Task III) and to enable the estimation of the variations in GWS using the estimated SWS and outputs of land surface models (Task IV).

### GRACE_TWS_

Three communal GRACE mascon solutions were utilized and reported relative to a 2004–2009 mean baseline. The first is the GRACE CSR-RL06M solutions provided by the University of Texas Center for Space Research (UT-CSR); the data provided are oversampled on an equiangular grid of size (0.25° × 0.25°)^77^. No post-processing and/or filtering or application of empirical scaling factors was applied^78^. The second is the mascon solutions from the Jet Propulsion Laboratory (JPL-RL06M), and the third is the spherical harmonic solution Rl06 version 4 from CSR (CSR-Rl06SH). The GRACE CSR-M solutions were derived using Tikhonov regularization with an L-ribbon approach to compute the regularization parameter and were resolved on an equal area geodesic grid of roughly 1° at the equator.

The seasonal variations in the GRACE_TWS_ time series were removed by adopting the following steps: (1) filling in the missing months of data using linear interpolation (gap-filled time series), (2) simultaneously fitting annual cycle components (sine and cosine) of the GRACE_TWS_ time series, and (3) removing the seasonal cycle from the non-gap-filled time series^43,79^. The non-seasonal GRACE_TWS_ time series were used to identify the periods during which the TEW experienced gains, losses, or maintained steady state conditions. The breakpoints between the investigated periods were identified using the regime shift detection (RSD) method^80^.

### Precipitation (GPCC)

The precipitation throughout a period of 100 years (1920–2020) over the TEW was derived from GPCC monthly satellite-gauge (51 rain gauge stations over the TEW) combined precipitation dataset. The full data monthly product with a spatial resolution of 2.5° is available through the GPCC server hosted by the Deutscher Wetterdienst (DWD), Offenbach, Germany^81^. The precipitation time series was reported in two ways: (1) the average monthly precipitation (2003–2020) over the TEW, and (2) the seasonal precipitation (1920–2020) by aggregating the monthly precipitation events that occurred during the wet season (November–April) and dry season (May–October) (Fig. 4). The wet season hereafter refers to the winter months when snow accumulates and the spring months when most of the accumulated snow melts, whereas the dry season refers to the summer months where minimal precipitation occurs. Seasonal precipitation hereafter refers to the summation of monthly precipitation during the wet season that extends from November of a particular year to April of the following year. Thus, the seasonal precipitation of 2019 refers to the summation of precipitation during the months of November and December in 2018 and the months of January through April of 2019. The dry season extends from May to October. In this respect, the AAP for a particular period hereafter refers to the total seasonal precipitation throughout the investigated period averaged over the TEW area. The GRACE_TWS_ and the GPCC time series were correlated to examine whether extreme seasonal precipitation events could have given rise to anomalously high GRACE_TWS_ values over the TEW.

The precipitation variability index (δ) was calculated using Eq. (1) to differentiate between the drought years (characterized by negative − δ values) and wet years (characterized by + δ values)^82^. A prolonged drought will be noted if a series of negative δ values were observed for consecutive years, and vice versa for a wet period, a series positive δ values.

1 $$\delta_{{\text{i}}} = \, \left( {{\text{P}}_{{{\text{i}} }} - \mu } \right)/\sigma ,$$

where δ_i_ is the precipitation variability index for a year (i), P_i_ the seasonal precipitation for a year (i), and µ and σ are the average and the standard deviation of the seasonal precipitation throughout the 1920–2020 time period, respectively. A prolonged drought will be noted if a series of negative δ values were observed for consecutive years, and vice versa for a wet period, a series positive δ values. The GRACE_TWS_ and the GPCC time series were correlated to examine whether extreme seasonal precipitation events could have given rise to anomalously high GRACE_TWS_ values over the TEW.

The potential climatic drivers for the AAP were investigated by comparing the monthly AAP to: (1) the MOI, (2) the ENSO index, (3) SST anomaly (SST-based index of SAO), (4) the NAO index, and (5) SST anomaly (SST-based index of NAO^83^). All climatic indices are presented as May–July anomalies.

### Surface water storage

We quantified the temporal variations in SWS_ALT_ across the TEW by measuring the variations in surface water elevation and in the areal extent of the main reservoirs and the natural lakes within the watershed. These reservoirs and lakes, 13 in number, include the Ataturk, Karakaya, and Keban reservoirs and Van Lake in Turkey; the Assad reservoir in Syria; the Tharthar, Mosul, and Raazza reservoirs and the Hammar 1, Hammar 2, Hammar 3, and Hammar 4 lakes in Iraq; and the Karkheh reservoir in Iran (Fig. 1). The maximum holding capacity of the investigated reservoirs (8 reservoirs) is 222 km^3^, which represents some 90% of the total holding capacity (250 km^3^) of all the dams within the watershed^58^.

The surface water levels time series was extracted from two main surface water data centers: (1) the Database for Hydrological Time Series of Inland Waters (DAHITI), which provides time series of water levels from multi-mission satellite radar altimetry^84^, and (2) the US Department of Agriculture Foreign Agricultural Service (USDAFAS) GRLM. The variations in surface water levels were estimated with respect to the temporal mean of the entire period (2003–2020). The monthly variations in the areal extent of the reservoirs and lakes were extracted from the Global Surface Water Explorer dataset (spatial resolution: 30 m)^85^ that was generated from multiple Landsat mission datasets (Landsat 5, 7, and 8), and in which each pixel was classified as a water or non-water pixel.

The following steps were implemented to estimate the temporal variations in the SWS_ALT_ for each of the investigated reservoirs and lakes. Variations in monthly surface water levels and in the areal extent of the reservoirs and lakes were extracted from radar altimetry and the Global Surface Water Explorer datasets, respectively. Because there were gaps in radar altimetry data over a few of the investigated reservoirs and lakes, linear regression relationships were derived where needed between surface water levels and areal extent of the individual reservoirs and lakes (Eq. 2)^86^, which were then used to estimate water levels for the months where radar altimetry data was absent.

2 $${\text{WL}}\left( {\text{t}} \right) \, = {\text{ AE }} \times {\text{ a}} + {\text{b,}}$$

where, WL(t) is the surface water level at time (t), AE is the areal extent of the reservoir or lake, and a and b are the slope and intercept, respectively.

The monthly time series for water levels and areal extent of the investigated reservoirs and lakes were then used to estimate the temporal variations in water volume for the investigated reservoirs and lakes, which were then summed up and the seasonal variations removed^87^ to extract non-seasonal SWS_ALT_ time series over the TEW in units of km^3^/month. The estimated SWS_ALT_ time series was used to calculate the rise or drop in surface water level for a particular year by subtracting from it the surface water level of the preceding year and using the peak surface water levels for each of the two consecutive years.

### GRACE groundwater storage

GRACE_TWS_ and outputs of the Global Land Data Assimilation System (GLDAS-2.1) NOAH-3.3 model were used to derive the variations in the groundwater storage compartment^88^. The NOAH-3.3 model provides a sum of soil moisture storage (SMS), canopy water storage (CWS) and snow water equivalent (SWE) but doesn’t account for SWS. The non-seasonal GRACE groundwater storage (GRACE_GWS_) time series was calculated by subtracting the simulated GLDAS storages (SMS, CWS, and SWE) and the SWS_ALT_ values from GRACE _TWS_ (Eqs. 3 and 4)^89^ and subsequent removal of the seasonal variations in the GRACE_GWS_. Similar (within 5%) values for GRACE_GWS_ were obtained if the seasonal cycle was removed from the GRACE_TWS_ and the GLDAS storages prior to subtracting the latter from the former.

3 $${\text{GRACE}}_{{{\text{TWS}}}} = {\text{ GRACE}}_{{{\text{GWS}}}} + \, \left( {{\text{SMS}} + {\text{SWE}} + {\text{CWS}}} \right)_{{{\text{GLDAS}}}} + {\text{ SWS}}_{{{\text{ALT}}}} ,$$

4 $${\text{GRACE}}_{{{\text{GWS}}}} = {\text{GRACE}}_{{{\text{TWS}}}} \, - \left( {{\text{SMS}} + {\text{SWE}} + {\text{CWS}}} \right)_{{{\text{GLDAS}}}} - {\text{ SWS}}_{{{\text{ALT}}}} .$$

### Uncertainty estimation

For GRACE_TWS_, the CSR-M-RL06 solutions were selected as the primary dataset for extracting trends over the investigated periods^79^, where the standard deviation between the three selected solutions (CSR-RL06M, JPL-RL06M, and CSR-Rl06SH) represents the uncertainty in the reported trend value^90^.

Errors associated with the calculated water mass trends for SWS_ALT_ were estimated using procedures described in Ref.^91^: (1) the residuals (R1) were calculated after removing the components of trend; (2) a 13 month moving average was applied to the calculated residuals (R1) to remove the remnant signal (e.g. interannual signal) and the residuals (R2) were estimated; (3) the standard deviation of R2 represented the upper limits of uncertainty (error in the monthly measurements) in time series; (4) Monte Carlo simulation techniques were performed by fitting trends and seasonal terms for many synthetic monthly datasets (n = 10,000), each with values chosen from a population of Gaussian-distributed numbers with standard deviation values similar to that of the examined population.

Errors in GLDAS combined component (SMS + SWE + CWS)_GLDAS_ simulations were calculated as the standard deviation between the three GLDAS version 2.1 land surface models (variable infiltration capacity (VIC), catchment land surface model (CLSM), and NOAH-3.3 simulations)^92^.

Finally, the standard deviation of the generated synthetic trends was interpreted as the trend error for the calculated water mass trends (e.g., SWS_ALT_ and (SMS + SWE + CWS)_GLDAS_). The trend errors in GRACE_GWS_ (σGWS) were calculated by adding, in quadrature, trend errors related to GRACE_TWS_, SWS_ALT_ and (SMS + SWE + CWS)_GLDAS_ trends (Eq. 5).

5 $${\mathrm{\sigma GWS}}_{\mathrm{GRACE}}=\sqrt{{{\mathrm{\sigma TWS}}^{2}}_{\mathrm{GRACE}}+{{\mathrm{\sigma SWS}}^{2}}_{\mathrm{ALT}}+{{\upsigma (\mathrm{SMS}+\mathrm{SWE}+\mathrm{CWS}) }^{2}}_{\mathrm{GLDAS}}}.$$

### Landsat images

The Landsat 8 satellite was launched in February 2013 to collect (spatial resolution: 15–100 m; scene size: 183 km east–west; 170 km north–south), temporal (revisit time: 16 days) global images in the visible, near-infrared, short-wave infrared, and thermal infrared wavelength regions 57. The temporal and spatial variations of Al-Huwaizah, Central, and Al-Hammar marshlands watershed were extracted from 30 m multispectral Landsat 8 data acquired in years 2017 and 2020.

### DEM from Shuttle Radar Topography Mission

A DEM from the SRTM covering the entire TEW was used to delineate the stream network and watershed boundaries using ArcGIS 10.8 hydrological tools.

## Supplementary Information

Supplementary Information.

## Data Availability

All data needed to evaluate the findings are provided in the manuscript and additional relevant datasets could be requested from the first author. The Global Reservoir and Lake Monitoring Database (GRLM; available at https://www.pecad.fas.usda.gov/cropexplorer/globalreservoir/). The Global Surface Water Explorer dataset (spatial resolution: 30 m; available at http://global-surface-water.appspot.com).
