# Supplementary material for: Buffering the impacts of extreme climate variability in the highly engineered Tigris Euphrates river system
Source: Sci Rep. 2022 Mar 9;12:4178. doi: 10.1038/s41598-022-07891-0 (PMC8907168; doi:10.1038/s41598-022-07891-0)
Supplement: Supplementary file 1 — Supplementary Information. [file 41598_2022_7891_MOESM1_ESM.pdf]

Supplementary Information for

Buffering the Impacts of Extreme Climate Variability in the Highly Engineered Tigris Euphrates River System

Karem Abdelmohsen[1,2], Mohamed Sultan[1]*, Himanshu Save[3], Abotalib Z. Abotalib[1,4], Eugene Yan[5], Khaled H. Zahran[2]

** corresponding author Email: Mohamed.sultan@wmich.edu*

I. Supplementary Figures

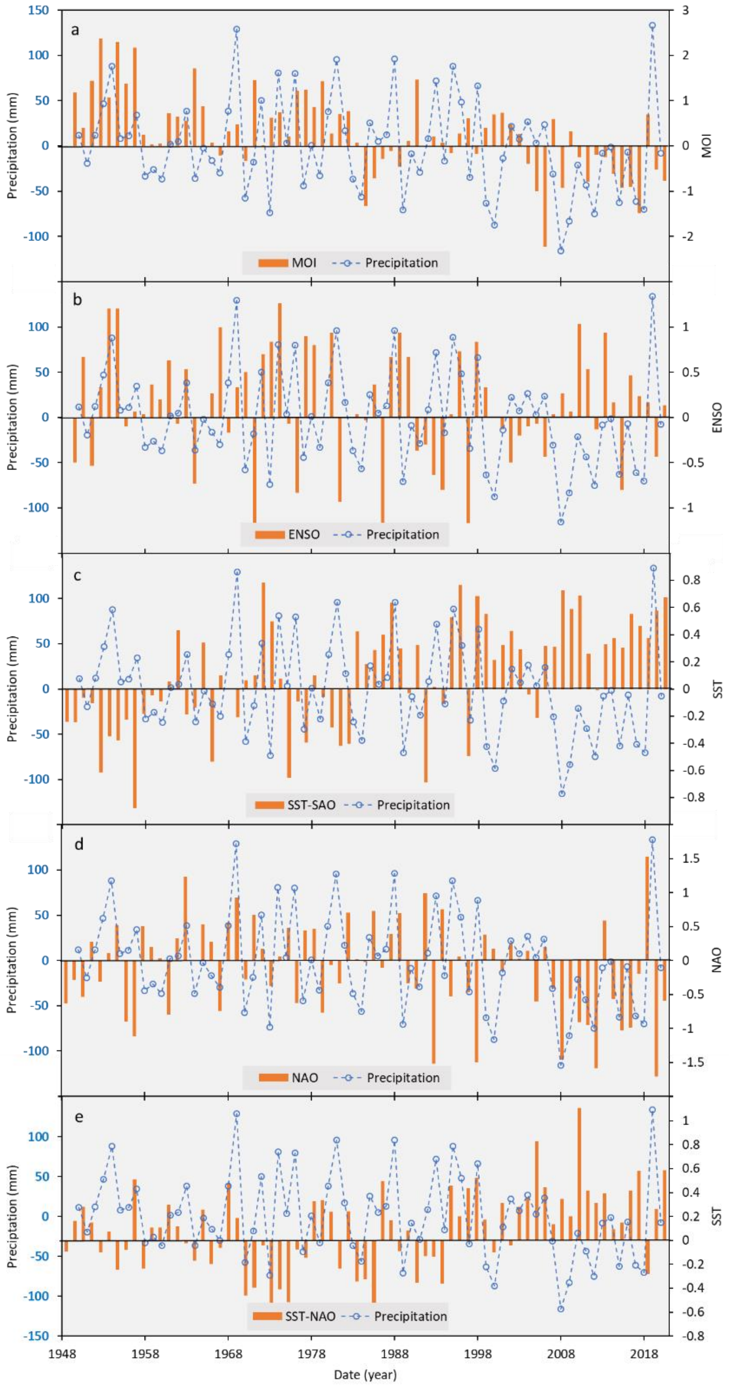

Supplementary Figure S1: Correlation between climatic oscillations and parameters and AAP over the TEW throughout the period 1950–2020. (a) AAP and MOI. **(b)** AAP and ENSO index. **(c)** AAP and SST (based on the SAO[1] index). **(d)** AAP and NAO index. **(e)** AAP and SST (based on the NAO[2] index).

II. Supplementary Notes

Historical and modern role of dams

Until recently, the State of Ceará in Brazil frequently suffered from severe droughts, but this situation was mitigated with the construction of large dams in the 1980s and 1990s, a development that made Ceará the premier state in water resource management in Brazil[3]. The construction of dams and complex engineering projects along the Pampanga River in the Philippines is enabling farm irrigation during both the rainy and the dry seasons and production of 30% of the country's rice crop[4]. The Aswan High Dam in Egypt, with its extensive storage capacity (162 km^3) regulates the entire irrigation system in the country, buffers the impact of the Nile Basin droughts[5], and mitigates the impacts of floods[6].

Climatic oscillations

The NAO the difference in pressure between two points (Azores and Iceland) over a time period across the North Atlantic Ocean controls the strength and direction of the westerlies between 30° and 60° latitude. A positive NAO allows strong westerly winds and wet winters in central Europe and drier winters in North Africa and the TEW, whereas a negative NAO index favors easterly winds with snowier winters in central Europe and wet winters and rainfall in southern Europe, North Africa, and the TEW[7,8]. The ENSO index (SOI) the difference in pressure between Tahiti and Darwin points in Australia is a measure of the large-scale fluctuations in air pressure between the western and eastern tropical Pacific. A positive ENSO (La Nina) brings drier winters with less precipitation over North Africa and the TEW, whereas a negative ENSO (El Nino) brings wet winters and precipitation over North Africa and the TEW[9]. The MOI the difference in sea level pressure between eastern and western Mediterranean affects the precipitation over Europe, North Africa, and the TEW. A positive MOI brings drier winters and less precipitation, whereas a negative MOI brings wet winters with added precipitation over these areas[8].

The SST-based indices of NAO and SAO represent the SST anomalies for the North Atlantic and Southern Atlantic Oceans, respectively[1], where the former (SST-NAO) is more dominant in determining winter temperature over the Middle East than SST-based index of ENSO[2]. The SST index is negatively correlated with precipitation, where the negative SST index implies more precipitation events during the winter[2].

III. Supplementary References

References

1. Christian, L., Philip J., W. & Matti, K. Impact of large-scale climatic oscillations on snowfall-related climate parameters in the world's major downhill ski areas: A review. *Mt. Res. Dev.* **32**, 431–445 (2012).
2. Cullen, H. M., Kaplan, A., Arkin, P. A. & DeMenocal, P. B. Impact of the North Atlantic Oscillation on Middle Eastern climate and streamflow. *Clim. Change* **55**, 315–338 (2002).
3. Gutiérrez, A. P. A., Engle, N. L., De Nys, E., Molejón, C. & Martins, E. S. Drought preparedness in Brazil. *Weather Clim. Extrem.* **3**, 95–106 (2014).
4. Gusyev, M. *et al.* Drought assessment in the Pampanga River basin, the Philippines–Part 1: Characterizing a role of dams in historical droughts with standardized indices. *21 Int. Congr. Model. Simul. (MODSIM), Novemb. 29–December 4, Queensland, Aust.* (2015).
5. Abdelhafez, A. A., Metwalley, S. M. & Abbas, H. H. 'Irrigation: Water Resources, Types and Common Problems in Egypt. in *Technological and Modern Irrigation Environment in Egypt*. (eds. Omran, E.E. and Negm, A.M.) 15–34 (Springer International Publishing, 2020).
6. Omran, E. S. E. and Negm, A. Environmental Impacts of the GERD Project on Egypt's Aswan High Dam Lake and Mitigation and Adaptation Options. in *Grand Ethiopian Renassance Dam versus Aswan High Dam.* (eds. Negm, A.M. and Abdel-Fattah, S.) 175–196 (Springer International Publishing, 2019).
7. Adamo, N., Al-Ansari, N., Sissakian, V. K., Laue, J. & Knutsson, S. The future of the Tigris and Euphrates water resources in view of climate change. *J. Earth Sci. Geotech. Eng.* **8**, 1792–9660 (2018).
8. Forootan, E. *et al.* Large-Scale Total Water Storage and Water Flux Changes over the Arid and Semiarid Parts of the Middle East from GRACE and Reanalysis Products. *Surv. Geophys.* **38**, 591–615 (2017).
9. Nazemosadat, M. J. & Cordery, I. On the relationships between ENSO and autumn rainfall in Iran. *Int. J. Climatol.* **20**, 47–61 (2000).
